# Neuronal Rubicon Represses Extracellular APP/Amyloid β Deposition in Alzheimer’s Disease

**DOI:** 10.3390/cells11121860

**Published:** 2022-06-07

**Authors:** Sandra Espinoza, Felipe Grunenwald, Wileidy Gomez, Felipe García, Lorena Abarzúa-Catalan, Sebastián Oyarce-Pezoa, Maria Fernanda Hernandez, Bastián I. Cortés, Markus Uhrig, Daniela P. Ponce, Claudia Durán-Aniotz, Claudio Hetz, Carol D. SanMartín, Victor H. Cornejo, Fernando Ezquer, Valentina Parra, Maria Isabel Behrens, Patricio A. Manque, Diego Rojas-Rivera, René L. Vidal, Ute Woehlbier, Melissa Nassif

**Affiliations:** 1 Laboratory of Neuroprotection and Autophagy, Faculty of Sciences, Universidad Mayor, Santiago 8580745, Chile; sc.espinozacerda@gmail.com (S.E.); wileidygomez20@gmail.com (W.G.); felipe.garciao@mayor.cl (F.G.); sebastian.oyarcep@mayor.cl (S.O.-P.); 2 Center for Integrative Biology, Faculty of Sciences, Universidad Mayor, Santiago 8580745, Chile; felipe.grunenwald@mayor.cl (F.G.); lorena.abarzua@gmail.com (L.A.-C.); fernandahernandezberrios@gmail.com (M.F.H.); bastian.cortes@mayor.cl (B.I.C.); patricio.manque@umayor.cl (P.A.M.); rene.vidal@umayor.cl (R.L.V.); ute.woehlbier@umayor.cl (U.W.); 3 Center for Regenerative Medicine, Facultad de Medicina Clínica Alemana, Universidad del Desarrollo, Santiago 7550000, Chile; markus.uhrig@web.de (M.U.); eezquer@udd.cl (F.E.); 4 Centro de Investigación Clínica Avanzada, Universidad de Chile, Santiago 8380456, Chile; dponcedelavega@gmail.com (D.P.P.); dazilsanmartin@gmail.com (C.D.S.); behrensl@uchile.cl (M.I.B.); 5 Center for Social and Cognitive Neuroscience, School of Psychology, Universidad Adolfo Ibañez, Santiago 7550313, Chile; claudia.duran@uai.cl; 6 Center for Geroscience, Brain Health, and Metabolism, Santiago 8380453, Chile; chetz@uchile.cl; 7 Program of Cellular and Molecular Biology, Institute of Biomedical Sciences, University of Chile, Santiago 8380453, Chile; vcornejocorona@ug.uchile.cl; 8 Buck Institute for Research on Aging, Novato, CA 94945, USA; 9 Departamento de Neurología y Neurocirugía, Hospital Clínico Universidad de Chile, Santiago 8380456, Chile; 10 Autophagy Research Center, Universidad de Chile, Santiago 8380456, Chile; vparra@ciq.uchile.cl; 11 Departamento de Bioquímica y Biología Molecular and Advanced Center for Chronic Diseases, Facultad de Ciencias Químicas y Farmacéuticas, Universidad de Chile, Santiago 8380456, Chile; 12 Departamento de Neurociencia, Facultad de Medicina Universidad de Chile, Santiago 8380456, Chile; 13 Clínica Alemana de Santiago, Universidad del Desarrollo, Santiago 13132, Chile; 14 Centro de Oncologia de Precision (COP), Escuela de Medicina, Universidad Mayor, Santiago 8580745, Chile; 15 Escuela de Biotecnología, Facultad de Ciencias, Universidad Mayor, Santiago 8580745, Chile; diego.rojas@umayor.cl; 16 Escuela de Tecnología Médica, Facultad de Ciencias de la Salud, Universidad Mayor, Santiago 8580745, Chile

**Keywords:** Alzheimer’s disease, autophagy, Rubicon, KIAA0226, RUBCN, APP, KIAA0226L, Pacer

## Abstract

Alzheimer’s disease (AD) is the most prevalent age-associated neurodegenerative disease. A decrease in autophagy during aging contributes to brain disorders by accumulating potentially toxic substrates in neurons. Rubicon is a well-established inhibitor of autophagy in all cells. However, Rubicon participates in different pathways depending on cell type, and little information is currently available on neuronal Rubicon’s role in the AD context. Here, we investigated the cell-specific expression of Rubicon in postmortem brain samples from AD patients and 5xFAD mice and its impact on amyloid β burden in vivo and neuroblastoma cells. Further, we assessed Rubicon levels in human-induced pluripotent stem cells (hiPSCs), derived from early-to-moderate AD and in postmortem samples from severe AD patients. We found increased Rubicon levels in AD-hiPSCs and postmortem samples and a notable Rubicon localization in neurons. In AD transgenic mice lacking Rubicon, we observed intensified amyloid β burden in the hippocampus and decreased Pacer and p62 levels. In APP-expressing neuroblastoma cells, increased APP/amyloid β secretion in the medium was found when Rubicon was absent, which was not observed in cells depleted of Atg5, essential for autophagy, or Rab27a, required for exosome secretion. Our results propose an uncharacterized role of Rubicon on APP/amyloid β homeostasis, in which neuronal Rubicon is a repressor of APP/amyloid β secretion, defining a new way to target AD and other similar diseases therapeutically.

## 1. Introduction

Alzheimer’s disease (AD) is the leading neurodegenerative disease associated with aging, affecting millions worldwide [1,2]. AD’s neuropathological hallmarks are the progressive accumulation of extracellular plaques containing amyloid-β peptides (amyloid β), intracellular neurofibrillary tangles (NFTs), atrophy of specific brain areas, and neuroinflammation. Cell and systems biology studies have reported disturbances in the autophagy and endolysosomal pathways as initial molecular changes in sporadic and familial AD cases, suggesting defects in these pathways underlying AD pathogenesis [3,4,5,6,7,8,9]. In line with these results, APP, the amyloid precursor protein, is a transmembrane protein situated in the cell membrane and several organelles, including endosomes and autophagic vesicles, indicating that failures in autophagy endolysosomal activities contribute to the amyloid β accumulation [9,10,11,12,13,14].

Autophagy is a conserved process that delivers cargo to the lysosome for degradation to maintain cellular homeostasis [15,16,17]. This process is particularly significant to neurons that must maintain their function during a lifetime [17,18,19,20]. Macroautophagy (hereafter referred to as autophagy) is the pathway that promotes the clearance of insoluble cargos, such as protein aggregates and dysfunctional organelles, by forming a double-membrane vesicle called autophagosome [15,21]. Autophagy-related (ATG) proteins tightly regulate the pathway [22,23,24,25,26]. One of the core protein complexes promoting autophagosome and endosome maturation includes the class III phosphoinositide 3-kinase (PI3KC3 or hVPS34), Beclin1, UVRAG, and Pacer [22,23,24]. Rubicon is the primary inhibitor of this complex at the last steps of autophagy and endocytosis [23,24,27]. Mechanistically, Rubicon antagonizes Pacer by binding to UVRAG, blocking Rab7 and PI3KC3 activation, inhibiting the autophagosome maturation and fusion with lysosomes [22,27]. Interestingly, decreasing autophagy activity during aging was correlated with rising Rubicon levels in tissues from worms, flies, and mice [28]. Furthermore, the knockdown of Rubicon in neurons was sufficient to increase lifespan in Drosophila, consistent with decreasing protein aggregates accumulation [28]. Recently, increased levels of Rubicon during aging were reported as a consequence of a gradual decline in a transcription factor called MondoA [29]. Age-related decline in MondoA is correlated with increased levels of Rubicon, mitochondrial impairment, and cellular senescence [29]. Rubicon and other ATG proteins, historically associated with the autophagy pathway, have been recently shown to perform essential roles in autophagy-independent processes. Rubicon, while being an inhibitor of autophagy activity, is required for LC3-associated phagocytosis (LAP) and LC3-associated endocytosis (LANDO), specific processes observed in phagocytic cells [30,31,32]. Rubicon-dependent LANDO in microglia was reported to contribute to the clearance of extracellular amyloid-β-containing plaques in a mouse disease model [31]. Previously, we found increased levels of Rubicon in the brain cortex from an AD mouse model [33]. However, the Rubicon cell-type expression in the central nervous system (CNS) and the potential contribution of a neuronal Rubicon to the amyloid β metabolism were not thoroughly studied in control or AD contexts.

To investigate the relevance of Rubicon in AD, here, we first examined Rubicon levels in human-induced pluripotent stem cells (hiPSCs), derived from an AD patient at an early-to-moderate stage of the disease. We then studied the cell-type localization of Rubicon in postmortem frontal cortex tissues from controls and severe AD patient donors, finding a marked neuronal localization. We also analyzed Rubicon cell-type localization in the hippocampus and brain cortex tissues from a transgenic mouse model of AD. To address the role of Rubicon in the progression of the disease, we generated an AD transgenic mouse model lacking Rubicon, evaluating autophagy markers, amyloid β burden, and neuronal cell death in the hippocampus, cerebral cortex, and amygdala tissues. 5xFAD mice lacking Rubicon showed specific changes in autophagy markers depending on the brain region evaluated. Rubicon deficiency led to increased amyloid β burden in the hippocampus without affecting neuronal death. To dissect the effect of Rubicon in a neuronal context, we depleted Rubicon in a neuroblastoma cell line expressing APP, finding a significantly increased release of APP to the cellular medium. Overall, our results uncovered a previously unanticipated role of the Rubicon in APP processing in neurons and AD, suggesting a dual effect on microglia, as was previously reported, and neurons on the amyloid β burden in AD.

## 2. Materials and Methods

### 2.1. Human-Induced Pluripotent Stem Cells (hiPSCs)

hiPSC from an AD patient and a non-dementia subject (Control) were generated from skin fibroblasts donated by Chilean patients from the Hospital Clínico de la Universidad de Chile (HCUCh). AD diagnosis was established according to the National Institute on Aging-Alzheimer’s Association workgroups criteria and the Clinical Dementia Rating (CDR) scale (0 to 3) [34,35]. The control donor took part in the same neurological and neuropsychological evaluations. Subjects gave written informed consent under the Declaration of Helsinki. The present study was carried out following the recommendations of the Ethics Committees of the HCUCh (HCUCh Human Subject Protocol Number #082) and from Universidad Mayor (#163/2020). We generated four hiPSC clones from a patient with early to moderate AD (female, 84 years old, CDR = 1) and five clones from a non-dementia subject as a control (female, 78 years old, CDR = 0). The karyotype of the hiPSCs clones was assessed at the Institute of Nutrition and Food Technology (INTA), Universidad de Chile. One of the AD clones presented chromosome alteration and was discarded. Fibroblasts were grown in MEMα media (Biological Industries, Kibbutz Beit-Haemek, Israel), with 10% FBS (Gibco, Thermo Fisher Scientific, Waltham, MA, USA) and 1% penicillin-streptomycin (Corning). They were reprogrammed using the Neon Transfection System (Thermo Fisher Scientific, Waltham, MA, USA). Cells were transfected with four plasmids in buffer R: pCXLE-hOCT3/4-shp53; Plasmid 2: pCXLE-hSK; Plasmid 3: pCXLE-hUL, and Plasmid 4: pCXWB-EBNA1 [36]. HiPSC-AD and hiPSC-control clones were grown in StemFlex medium (Gibco, Thermo Fisher Scientific, Waltham, MA, USA) on plates with a support matrix (Matrigel), following [37]. The ROCK Inhibitor Y-27632 10 µM (Biorbyt, Cambridge, UK) was used during division to improve cell survival and reduce selective pressure. The StemFlex medium was changed every two days until reaching 70–80% confluence. Pluripotency markers (SOX2 and OCT4) were checked by quantitative real-time PCR (RT-qPCR) and immunofluorescence (IF) (Supplementary Material and Methods and Supplementary Figure S1a–c).

### 2.2. Human Tissues

Paraffin-embedded samples from human postmortem brain tissues were a kind donation from the Knight AD Research Center (Knight ADRC) from the Washington University in St. Louis, MO, USA [38]. Briefly, 10 µm samples from the frontal cortex of AD patients and non-dementia control (Control) subjects were obtained from Knight ADRC under the approved Institutional Ethical Review Board protocol (#T1011). The operational criteria for classifying AD and other pathologies defined by the National Alzheimer Coordinating Center (NACC) were applied. The diagnosis was determined using consensus neuropathologic criteria for AD and non-AD disorders [34,35]. The CDR was applied to assess dementia severity. Braak stages analysis in postmortem samples confirmed severe AD diagnosis [39]. Braak stages I/II indicate NFTs limited to the entorhinal region of the brain, Braak stages III/IV indicate involvement of limbic regions such as the hippocampus, and Braak stages V/VI indicate moderate-to-severe neocortical involvement [40]. Postmortem tissues were selected from four healthy controls (CDR = 0; Braak stages I and II) and four severe AD patients (CDR = 3), with a Braak stage IV with six to ten years of disease duration (Table 1).

### 2.3. Transgenic Mice

The Rubicon knockout (KO or Rub−/−) mice were generated by CRISPR/cas9 as previously described [30] and kindly provided by Dr. Douglas R. Green from St. Jude Children’s Research Hospital, Memphis, TN, USA (C57BL/6 background). As an AD model, we used 5xFAD transgenic mice obtained from Jackson’s laboratory (B6SJL-Tg, APPSwFlLon, PSEN1*M146L*L286V/6799Vas), which overexpress mutant human APP with the Swedish, Florida, and London familial AD (FAD) mutations along with human PS1 harboring two FAD mutations, at a C57BL/6xSJL genetic background [40,41]. Male 5xFAD mice were backcrossed for several generations to C57BL/6 wild-type females to obtain a line with a >90% C57BL/6 genetic background before crossing with Rubicon knockout mice, similar to described in [41]. As such, 5xFAD males were crossed with female Rubicon heterozygotes (F1), generating 5xFAD/Rub+/− animals. Next, 5xFAD/Rub+/− (F2) mice were crossed to obtain four genotypes: WT (WT/Rub^+/+^), Rubicon KO (WT/ Rub−/−), 5xFAD (5xFAD/Rub+/+), and Rubicon-KO/5xFAD (5xFAD/Rub−/−) animals. Only the F2 generation was used for experimental assessment and characterization. The animals were viable and born with an expected Mendelian rate. Further, 5xFAD mice wild type or lacking Rubicon were analyzed at six months in a sex and age-matched way. Animals were genotyped with conventional PCR (Supplementary Material and Methods and Supplementary Figure S2a) and further checked by Western blot and IF assays (Supplementary Figure S2b,c). The work with animals followed the recent version of the Animal Research: Reporting In Vivo Experiments (ARRIVE) guidelines and was approved by the Bioethical and Biosecurity Committee from Universidad Mayor, closely monitored by a veterinarian responsible for the mice’s well-being.

### 2.4. Mouse Tissue Preparation

Mice were euthanized at six months by isoflurane inhalation, and brains were collected. After perfusion with PBS1X and dissection, the right hemibrain of each animal was fixed in paraformaldehyde (PFA, Merck KGaA, Darmstadt, Germany, 30525-89-4) 4% for 24 h and embedded in paraffin as previously reported [33,42]. The left hemibrain was saved at −80 °C for biochemical analysis. Paraffin-embedded brains were processed and sequentially sectioned coronally at 10 µm, from lambda 0 to −4.5 mm, using the Leica RM2125 RTS Thermo Scientific HM325 microtome (Thermo Fisher Scientific, Waltham, MA, USA) and were collected in positively charged slides. The slides were deparaffinized with xylol and hydrated with descending alcohol concentrations until reaching distilled water. Three to five anteroposterior tissue sections per animal/staining were used for image analysis quantifications.

### 2.5. Histology Analysis

Paraffin-embedded brain samples from human subjects and transgenic mice were analyzed using the same procedures below. For IF, epitopes were exposed with citrate buffer at 96 °C for 1 h. As previously described [33,42], slides were washed three times with 0.05% Tween20 in PBS1X. After blocking non-specific binding sites, slices were incubated with primary antibodies diluted in 3% BSA and 0.05% Tween20 in PBS1X solution overnight at 4 °C. Primary antibodies used: anti-Rubicon (rabbit, 1:1000, Thermo Fisher Scientific, Waltham, MA, USA, PA5-38017); anti-GFAP (mouse, 1:1000, Merck KGaA, Darmstadt, Germany, MAB360); anti-NeuN (mouse, 1:1000, Merck KGaA, Darmstadt, Germany, MAB377); and, for activated microglia, anti-Iba1 (rabbit, 1:750, FUJIFILM Wako Chemicals, Hong Kong, 019-19741). Secondary antibodies used were anti-rabbit Alexa 555, anti-mouse Alexa 488 (both 1:500, Thermo Fisher Scientific, Waltham, MA, USA, A21428 and A11001, respectively), and DAPI (1:10000, Thermo Fisher Scientific, Waltham, MA, USA, D1306) was employed as a nucleus marker. For amyloid β deposition analysis, brain samples were exposed to 70% formic acid for 20 min. Blocking was performed with 3% BSA in PBS 1X Tween20 0.02% for 1 hr. Primary antibody 4G8 (mouse, 1:1000, Biolegend, San Diego, CA, USA, 800701) was incubated overnight at 4 °C. Alexa 555 secondary antibody (mouse, 1:1000, Thermo Fisher Scientific, Waltham, MA, USA, A28180) and DAPI were incubated for two hours. Negative controls with secondary antibodies for human samples are presented in Supplementary Figure S3. Finally, sections were coverslipped with Fluoromont mounting medium (Thermo Fisher Scientific, Waltham, MA, USA) for microscopy analysis.

### 2.6. Cell Culture Experiments

Neuroblastoma Neuro2A cells were purchased (ATCC, Manassas, VA, USA) and cultured in Dulbecco’s modified Eagles medium (DMEM) supplemented with 5% fetal bovine serum (FBS) (Life Technologies, Thermo Fisher Scientific, Waltham, MA, USA). Neuro2A APP stable cell line was generated by transfection of the EGFP-tagged APP construct (kindly provided by Dr. Patricia Burgos, [43]) with Lipofectamine 2000 (Invitrogen, Thermo Fisher Scientific, Waltham, MA, USA) according to the manufacturer’s instructions and maintained at 0.5 mg/mL of G418. For loss-of-function experiments, Neuro2A-APP cells were seeded at a concentration of 2 × 10^5^ cells/well in six-well plates and transfected 24 h later with ON-TARGETplus smart-pool siRNAs targeting mouse Rubicon (si*Rub*), mouse Atg5 (si*Atg5*), mouse Rab27A (si*Rab27A*), and ON-TARGETplus non-targeting siRNAs as a control (si*Ctrl*) (all purchased from, Dharmacon, Lafayette, CO, USA) using Dharmafect Transfection Reagents (Dharmacon, Lafayette, CO, USA, T-2001-01). Briefly, 4 μL Dharmafect was used to obtain a final concentration of 20 nM siRNA/well. After 48 h of transfection, cell biology experiments were performed. For autophagy flux experiments, Neuro2A cells were treated with bafilomycin A1 (BAF, 25 nM) and Chloroquine (CQ, 25 µM) for the indicated time. Then, total protein extracts were generated, and LC3-II was analyzed by Western blot.

### 2.7. Images Analysis

All images of the previously described IFs were obtained using the Leica DMI8 inverted microscope and ImageJ/Fiji (NIH, Bethesda, MD, USA) software to quantify intensity. Final representative images were acquired with a Leica TCS SP8 laser scanning inverted confocal microscope and LAS X software, using the 10×, 40×, and 60× objectives. ImageJ/Fiji software was used to process stacked images.

### 2.8. RNA Extraction and Quantitative Real-Time PCR

The murine brain cortex and hippocampus were separated by dissecting on ice for biochemical assays. Homogenization was performed with sterile PBS1X (Gibco, Thermo Fisher Scientific, Waltham, MA, USA), RIPA1X, and EDTA-free protease inhibitor cocktail (Thermo Fisher Scientific, Waltham, MA, USA). Total RNA was extracted by first homogenizing tissues in PBS1X with protease inhibitors (Thermo Fisher Scientific, Waltham, MA, USA, A32955) in a weight-per-volume ratio of 1:1. For hiPSC and mice tissue extracts, total RNA was extracted using TRIzol-LS (Invitrogen, Thermo Fisher Scientific, Waltham, MA, USA, 10296028) following the manufacturer’s protocol and quantified by NanoDrop Spectrophotometer (NanoDrop 2000c, Thermo Fisher Scientific, Waltham, MA, USA). cDNAs were synthesized using the iScript™ Reverse Transcription Supermix for RT-qPCR (Bio-Rad Laboratories, Hercules, CA, USA). RT-qPCR was carried out via SYBR Green assays (SsoAdvanced™ Universal SYBR^®^ Green Supermix, Bio-Rad Laboratories, Hercules, CA, USA) using the RT-qPCR System (CFX96 Touch Real-Time PCR Detection System, Bio-Rad Laboratories, Hercules, CA, USA). Each sample was run in triplicate. Data for each transcript were normalized to 18S rRNA (human) and β-actin (mice) as internal controls. Transcript levels were quantified by using the ΔΔCt value method. For hiPSCs, primers used were as follows: POU5F1 5′-GATCACCCTGGGATATACAC-3′ and 5′-GCTTTGCATATCTCCTGAAG-3′; SOX2 5′-ATAATAACAATCATCGGCGG-3′ and 5′-AAAAAGAGAGAGGCAAACTG-3′; RCAN1.4 5′-CTCACTAGGGGCTTGACTGC-3′ and 5′-CAGGCAATCAGGGAGCTAAA-3′; human RUBICON 5′-CTGGCAGTTCGTGAAAGACA-3′ and 5′-TTAGCAGGAAGGCAGCATCT-3′; 18S 5′-GATATGCTCATGTGGTGTTG-3′ and 5′-AATCTTCAGTCGCTCCA-3′. For murine samples, primers were: mouse Rubicon 5′-TTCAGCATCTCCGAGTCCTT-3′ and 5′-AATCCCGTGAACTGAACTGG-3′; Atg5: 5′-GCC TAT ATG TAC TGC TTC ATC CA-3′ and 5′-CAT TTC AGG GGT GTG CCT TCA-3′; Rab27A 5′-TCG GAT GGA GAT TAC GAT TCA CT-3′ and 5′-TCC CTG AAA ATG CCC ACT-3′; TNF-α 5′-GGTCTGGGCCATAGAACTGA and 5′-CAGCCTCTTCTCATTCCTGC-3′; IL-6 5′- TGGTACTCCAGAAGACCAGAGG-3′ and 5′-AACGATGATGCAGCACTTGCAGA-3; TGF-β1 5′-CACTGATACGCCTGAGTG-3′ and 5′-GTGAGCGCTGAATCGAAA-3′, and β-actin 5′-AAGATCATTGCTCCTCCTGA-3′ and 5′-TACTCCTGCTTGCTGATCCA-3′.

### 2.9. Western Blot and Dot Plot Analysis

Samples from hiPSCs, mouse tissues, and Neuro2A cells were collected and homogenized in RIPA 1X in PBS1X lysis buffer (150 mM NaCl, 50 mM Tris, 1% TritonX-100, 0.1% SDS, 1% DOC) containing protease inhibitor cocktail (Thermo Fisher Scientific, Waltham, MA, USA). Protein concentration was determined by BCA assay (Pierce, Thermo Fisher Scientific, Waltham, MA, USA, or Dual-Range™ BCA Protein Assay Kit, Visual Protein). For the extracellular release of APP species evaluation, Neuro2A APP-EGFP-conditioned media were collected 24, 48, and 72 h after plating the cells and centrifuged at 2500 rpm. The supernatant was subjected to vacuum filtration through a 96-well dot blot apparatus (Bio-Rad Laboratories, Hercules, CA, USA) containing a PVDF membrane, similar to [44]. Membranes were blocked using Tris-buffered saline, pH 7.6, containing 0.1% (*v*/*v*) Tween 20 (TBST) buffer containing 5% milk, and incubated with primary antibody at 4 °C overnight. Antibodies and dilutions used were: anti-Rubicon (rabbit, 1:1000, Cell Signaling Technology, Danvers, MA, USA, 8465), anti-RCAN1.1 (rabbit, 1:3000, Sigma-Aldrich, Merck KGaA, Darmstadt, Germany, D6694), anti-SQSTM1/p62 (rabbit, 1:10000, Abcam, Cambridge, UK, ab91526), anti-Pacer (mouse, 1:1000, Abmart, Shanghai, CN, 1H/11), anti-Beclin1 (rabbit, 1:1000, Santa Cruz Biotechnology, Dallas, TX, USA, sc-11427), anti-LC3 (rabbit, 1:1000, Cell Signaling Technology, Danvers, MA, USA, 3868), anti-Atg5 (rabbit, 1:1000, Cell Signaling Technology, Danvers, MA, USA, 2630), and anti-amyloid β 1–16 (mouse, 1:1000, Biolegend, San Diego, CA, USA, 6E10 SIG-39320). β-Actin (rabbit, 1:1000, Cell Signaling Technology, Danvers, MA, USA, 4967), GAPDH (mouse, 1:1000, Santa Cruz Biotechnology, Dallas, TX, USA, sc-365062), and α-Tubulin (rabbit, 1:1000, Cell Signaling Technology, Danvers, MA, USA, 4967) were used as loading controls. Secondary HRP-conjugated anti-rabbit (1:3000, Jackson ImmunoResearch, West Grove, PA, USA, 711-035-152) or anti-mouse (1:3000, Jackson ImmunoResearch, West Grove, PA, USA, 715-035-150) were used. Bands were detected using ECL and quantified by scanning densitometry (ChemiDoc XRS+ system; Bio-Rad Laboratories, Hercules, CA, USA). Densitometric analysis was performed using ImageJ/Fiji software. 

### 2.10. Statistical Analysis

The statistical analysis used to compare the expression by RT-qPCR and Western blot of each gene between groups was performed using the non-parametric Mann–Whitney test. For IF assays of tissues derived from 5xFAD mice, data were analyzed using a two-way ANOVA followed by Sidak’s multiple comparisons test. For IF of human tissue samples, data were analyzed using the Mann-Whitney test. All values are expressed as the mean ± SEM. All statistical analyses were performed using GraphPad Prism 8 software. A value of *p* < 0.05 was considered statistically significant for all measures.

## 3. Results

### 3.1. Levels of Rubicon Are Increased in hiPSCs from an AD Patient from Early-to-Moderate Stage

**Figure 1:**
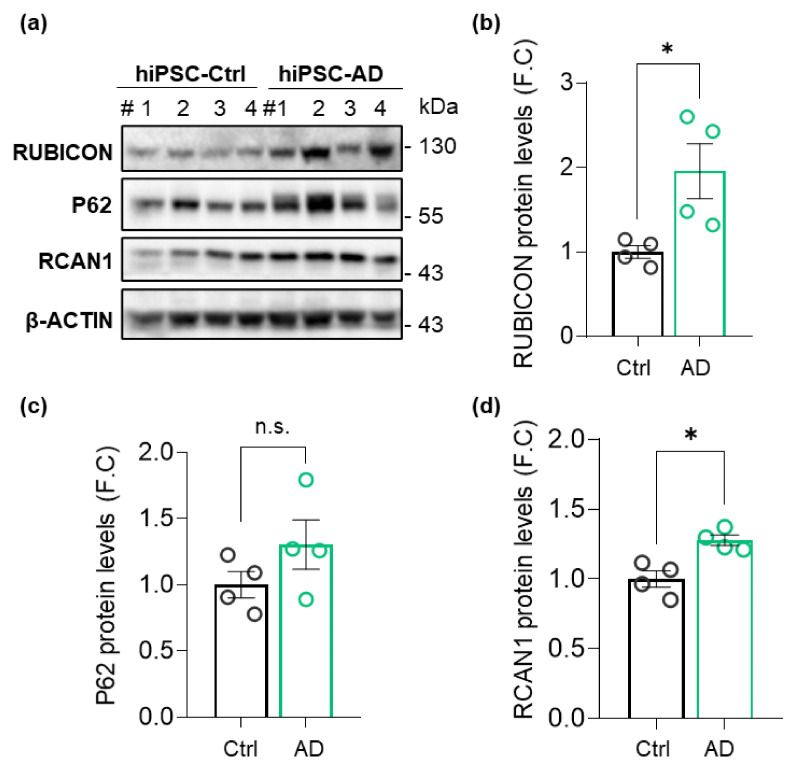
RUBICON protein levels are increased in human-induced pluripotent cells (hiPSCs) derived from an AD patient. (**a**) Total protein extracts were prepared from control (Ctrl) and AD hiPSCs. RUBICON, P62, RCAN1, and β-ACTIN levels were determined by Western blot, *n* = 4. Protein content was normalized with β-ACTIN. #, number of independent cell clones. (**b**) Densitometric analysis of normalized RUBICON, (**c**) P62, and (**d**). RCAN1 levels from total extracts. FC, fold change. Data are mean ± SEM (*n* = 4). * *p* < 0.05 as determined by a Mann-Whitney *t*-test, n.s., no significance.

To investigate the potential relevance of Rubicon at the earliest stages of AD progression in patients, we assessed Rubicon levels in hiPSCs generated from a sporadic AD patient and an age and sex-matched non-dementia donor (Ctrl). hiPSCs from AD patients have allowed the study of pathogenic mechanisms at the early or middle stages of the disease, which can accelerate findings and assist in interpreting results from postmortem tissue analysis from advanced stages [45,46,47]. Control and AD hiPSC lines expressed SOX2 and OCT4 pluripotency markers, as evaluated by IF and RT-qPCR (Supplementary Figure S1a–c). Western blot assay showed a significant increase in Rubicon protein levels in hiPSC-derived clones from AD compared to control (Figure 1a,b), with no significant difference detected at the transcriptional level (Supplementary Figure S1d). P62/SQSTM1 (P62) levels, an autophagy receptor protein cleared by autophagy with its substrates [48], were not significantly different between groups (Figure 1a,c). We also evaluated a protein that was overexpressed in AD [49], the regulator of the Calcineurin 1 gene (RCAN1). RCAN1 levels were increased in hiPSC-AD (Figure 1a,d) compared to controls. Hence, we found an increase in Rubicon protein levels in hiPSCs from an early-to-moderate-stage AD patient.

### 3.2. Rubicon Is Located in Neurons, and Its Levels Are Increased in Postmortem Brain Samples from AD Patients

**Figure 2:**
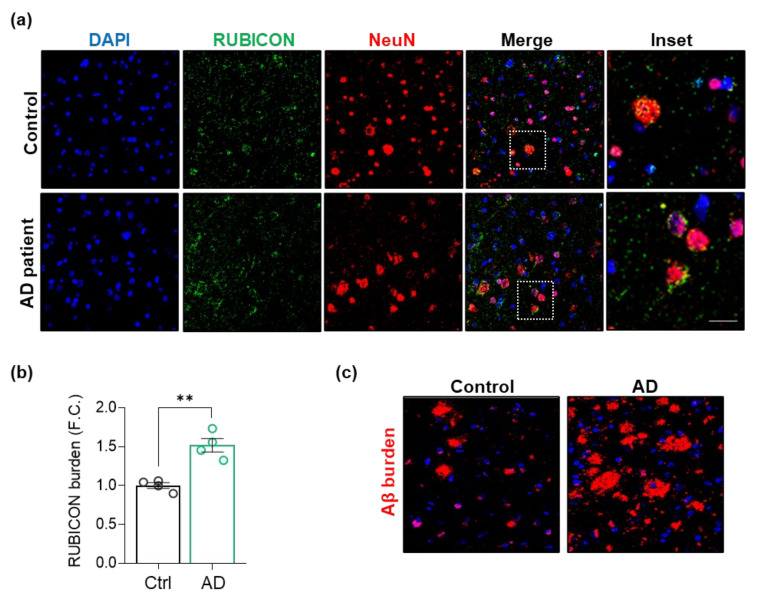
RUBICON is located in neurons, and its levels are increased in postmortem sections of the frontal cortex from AD patients. (**a**) Z-stack of confocal images showing detection of RUBICON (green), NeuN (red), and nuclei stained with DAPI (blue). Images represent controls (upper row, *n* = 4) and AD patients (lower row, *n* = 4). Scale bar: 50 μm. (**b**) Quantification of the intensity of RUBICON fluorescence in sections of four AD patients and four healthy controls. FC, fold change. (**c**) Representative Z-stack of confocal immunocytochemistry images detecting amyloid β plaques (4G8 antibody, red) and nucleus (DAPI, blue) from control and AD patients’ frontal cortex sections. Scale bar: 50 μm. Data are mean ± SEM. ** *p* < 0.01 as determined by a Mann-Whitney *t*-test.

Rubicon is an inhibitor of canonical autophagy and endocytosis activity [23,24]. In contrast, it is an essential component for specific LAP and LANDO processes, specifically in phagocytic cells, such as microglia [30,31]. To dissect the role of the Rubicon in CNS and its relevance to AD, we investigated its cell-type localization under physiological and disease conditions in human and murine CNS samples. First, we evaluated the localization of Rubicon in postmortem frontal brain cortex tissues from severe AD patients (CDR = 3 and Braak IV) and non-dementia control subjects (Table 1, Figure 2). Rubicon was reported to locate in late endosomes and lysosomes, presenting a vesicular profile in cellular IFs [23,24,50,51]. In human CNS samples, Rubicon showed a vesicular disposition in control and AD patients, showing a perinuclear profile (Figure 2a), markedly present in cells positive to neuronal marker NeuN (Figure 2a, and negative control in Supplementary Figure S3). We observed Rubicon staining to NeuN-negative cells as well. Interestingly, we detected an increased staining intensity of Rubicon in AD samples compared to controls in frontal cortex tissues (Figure 2b). Figure 2c shows representative amyloid β staining in samples from severe AD patients and controls. Our results show that Rubicon presents a marked localization in human brain cortex neurons and increased staining in AD patient samples compared to non-dementia donors. A clear vesicular profile of Rubicon was observed in human samples, as previously shown by its localization in late endosomes and lysosomes [23,24,50,51].

**Figure 3:**
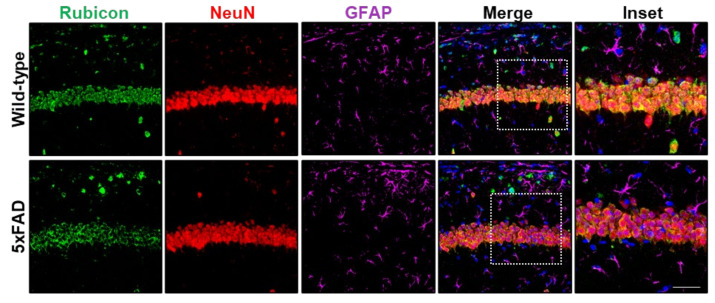
Rubicon is detected in neurons in hippocampal sections of wild-type and 5xFAD mice at the symptomatic stage (6 months old). Z-stack of confocal images detecting Rubicon (green), the neuron marker NeuN (red), and the astrocytic marker GFAP (purple) by immunofluorescence. Nuclei are stained with DAPI. Images are representative of at least four animals for each genotype. Scale bar: 50 μm, Inset: Scale bar: 20 μm.

We and others have shown increased levels of Rubicon in the cerebral cortex of AD transgenic mice by Western blot [33,52]. We also reported a Rubicon neuronal profile in the spinal cord of the amyotrophic lateral sclerosis (ALS) SOD1-mutant mice model [42]. Here, we performed IF staining and confocal microscopy assays of serial brain cortex and hippocampus sections of wild-type and age-matched littermate 5xFAD mice at the symptomatic stage (six months). Rubicon was expressed in NeuN-positive neurons in controls and 5xFAD hippocampi samples and in some GFAP-positive astrocytes (Figure 3 and Figure S4a,b). These results were similar in the mouse brain cortex (Supplementary Figure S4c,d). However, Rubicon was also expressed in NeuN- or GFAP-negative cells. These observations are similar to what was previously reported in the CNS of APP/PSEN1 mice [52]. Hence, our results show that Rubicon is highly expressed in neuronal cells in the human and murine CNS, with a rise in its expression in postmortem frontal cortex samples from severe AD cases.

### 3.3. 5xFAD Mice Lacking Rubicon Present Amyloid β Accumulation in the Hippocampus

**Figure 4:**
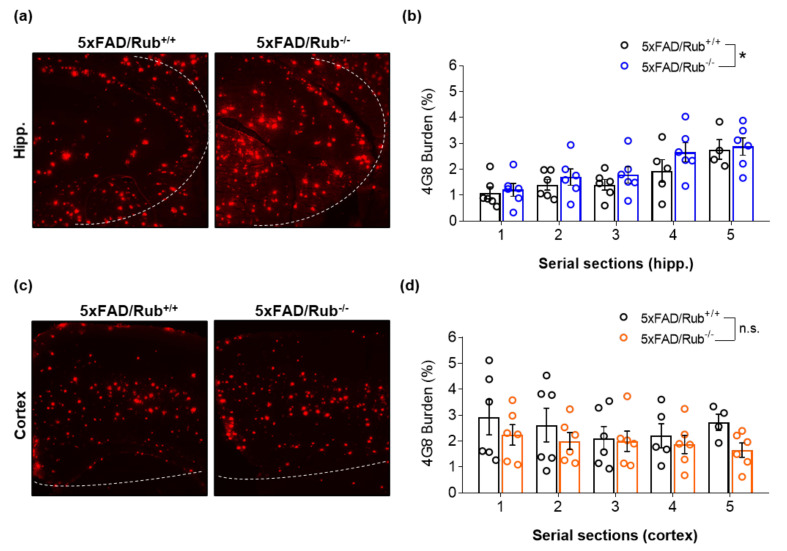
Amyloid β burden is increased in hippocampus sections from 5xFAD-lacking Rubicon mice at the symptomatic stage but not in the cortex. (**a**) Representative Z-stack of immunocytochemical confocal images of amyloid β plaque detection (4G8, red) in the hippocampus and cortex (**c**) of 5xFAD mice with regular expression of Rubicon (5xFAD/Rub+/+) on the left and mice lacking Rubicon (5xFAD/Rub−/−). Images are representative of at least four animals for each genotype. (**b**,**d**) Quantification of the intensity of fluorescence of 4G8 burden in serial sections of the hippocampus (**c**) (from (**a**)) and cortex (**d**) (from (**b**)). Data are mean ± SEM. * *p* < 0.05 as determined by repeated-measures ANOVA, n.s., no significance.

Given the importance of autophagy to the CNS and age-related diseases, such as AD, we hypothesized that reducing Rubicon levels would stimulate autophagy, improve spacial memory, and reduce amyloid β accumulation in mice. Here, we generated an AD transgenic mouse model lacking Rubicon by crossbreeding 5xFAD mice with Rubicon KO mice to investigate this possibility. The loss of Rubicon in 5xFAD/Rub−/− was confirmed by RT-qPCR, Western blot, and IF analysis (Supplementary Figure S2). We then evaluated the amyloid β burden in CNS regions affected in AD, the hippocampus, brain cortex, and basolateral amygdala from experimental groups at six months of age, when amyloid β plaques are markedly present in CNS from 5xFAD mice [40,41]. To avoid a partial evaluation of amyloid β accumulation in only one slide from CNS regions, we performed serial sections from experimental mice’s hippocampus and brain cortex. We observed a significant increase in amyloid β burden in the hippocampus of 5xFAD/Rub−/− mice (Figure 4a,b), which was not replicated in brain cortex sections (Figure 4c,d), or a region affected lately in AD, basolateral amygdala (Supplementary Figure S5a,b). Thus, Rubicon’s effects on amyloid β burden depend on the evaluated CNS region, presenting a more prominent effect on the hippocampus of 5xFAD mice.

### 3.4. Neuronal Loss, Astrogliosis, and Microglial Activation in 5xFAD Lacking Rubicon

**Figure 5:**
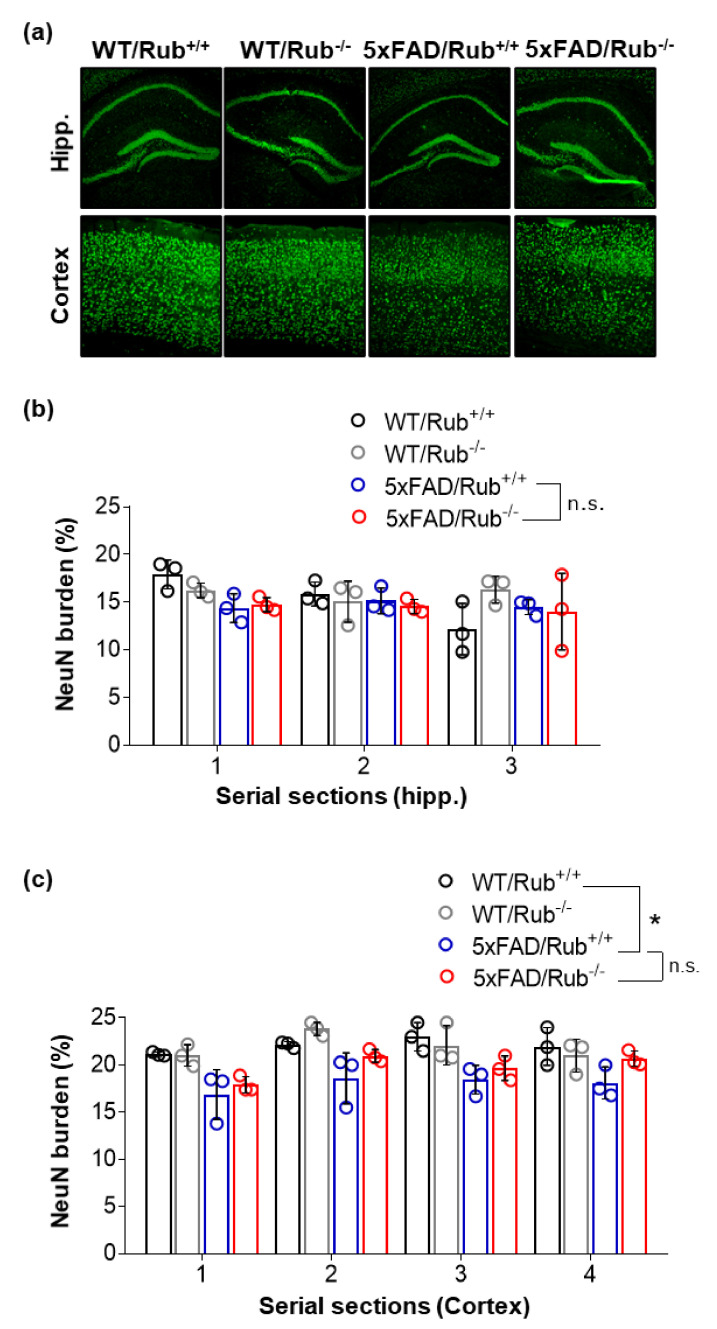
The lack of Rubicon in 5xFAD mice does not change the number of neurons in serial sections of the hippocampus and brain cortex tissues. (**a**) Representative Z-stack of confocal images of immunocytochemistry for the detection of neurons (NeuN, green) in the hippocampus (upper layer) and cortex (lower layer) of wild-type mice with and without regular expression of Rubicon (WT/Rub+/+; WT/Rub−/−) and 5xFAD mice with and without expression of Rubicon (5xFAD/Rub+/+; 5xFAD/Rub−/−). (**b**,**c**) Quantification of the intensity of fluorescence of neuronal marker NeuN in serial sections of the hippocampus (**b**) and cortex (**c**) of four genotypes analyzed. Data are mean ± SEM. * *p* < 0.05 as determined by repeated-measures ANOVA, n.s., no significance.

Next, we investigated if 5xFAD animals lacking Rubicon present changes in the number of the hippocampus and brain cortex neurons. We did not observe a hippocampal neuronal loss in 5xFAD/Rub+/+ mice compared to wild-type animals (Figure 5a,b), as previously reported for this 5xFAD mouse model background [41], and Rubicon deficiency did not change this phenotype (Figure 5a,b and Supplementary Figure S6). Although 5xFAD/Rub+/+ mice showed impairment in a cognitive test associated with hippocampus-dependent memory (Morris water maze test—MWM) compared to controls, there were no differences between 5xFAD animal groups (Supplementary Figure S7a). MWM assay also revealed that 5xFAD animals traveled a similar distance between Rubicon genotypes, showing no apparent motor performance differences (Supplementary Figure S7b).

We detected decreased neuron number in both 5xFAD transgenic mouse groups in the brain cortex compared to wild-type controls (Figure 5c). However, there were no significant differences due to the loss of Rubicon expression (Figure 5a,c).

**Figure 6:**
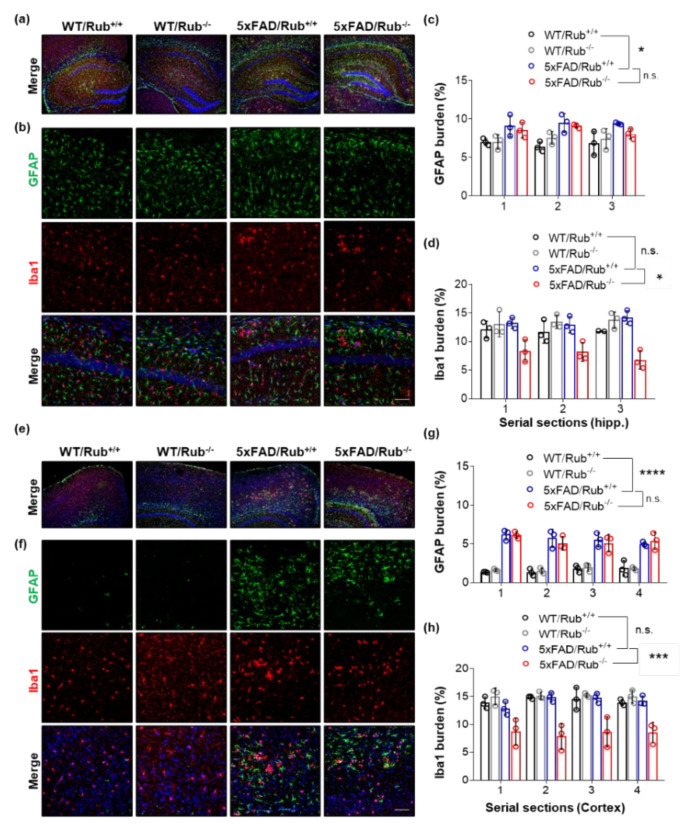
The lack of Rubicon in 5xFAD mice reduces the microglial marker in hippocampus and brain cortex serial sections. (**a**,**b**,**e**,**f**). Representative Z-stack of confocal images of immunocytochemistry for the detection of astrocytes (GFAP, green), microglia (Iba1, red), and nucleus (DAPI, blue) in serial sections of the hippocampus (**a**,**b**) and cortex (**e**,**f**) of wild-type mice with and without regular expression of Rubicon (WT/Rub+/+; WT/Rub−/−) and 5xFAD mice with and without expression of Rubicon (5xFAD/Rub+/+; 5xFAD/Rub−/−). Quantification of the fluorescence intensity of astrocytic marker GFAP ((**c**), hippocampus or (**g**), cortex) and microglial marker Iba1 ((**d**), hippocampus or (**h**), cortex) in serial sections of the hippocampus of four genotypes analyzed. (**b**,**f**), scale bar: 50 μm. Data are mean ± SEM. * *p* < 0.05; *** *p* < 0.001; **** *p* < 0.0001 as determined by repeated-measures ANOVA, n.s., no significance.

**Figure 7:**
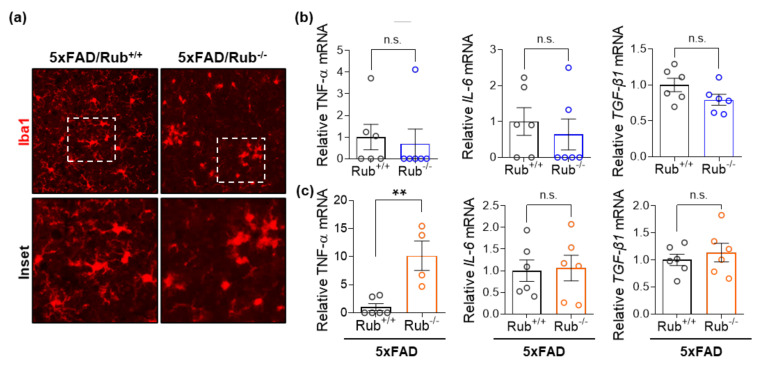
Microglial cells in the cortex from 5xFAD mice lacking Rubicon are more activated. (**a**) Representative Z-stack of confocal images of immunocytochemistry for the detection of microglia (Iba1, red) in the cortex and a zoom of the image (white square, inset) were obtained from 5xFAD mice with and without expression of Rubicon (5xFAD/Rub+/+; 5xFAD/Rub−/−). (**b**,**c**) Transcript expression of cytokines TNF-α, IL-6, and TGF-β1 was measured in samples from the hippocampus (**b**) and brain cortex and (**c**) of 5xFAD/Rub+/+ and 5xFAD/Rub−/− mice at six months of age. Data are mean ± SEM. ** *p* < 0.01 as determined by the Mann-Whitney *t*-test, n.s., no significance.

In a recent study, researchers reported neuronal death in 5xFAD deficient in Rubicon associated with reactive microglial activation and pro-inflammatory cytokine production [31]. We then investigated astrocytosis and microglial activation in our experimental animals. While we observed an increase in astrocytes in 5xFAD compared to wild-type experimental animals, we found no differences in astrogliosis between 5xFAD genotypes in the hippocampus (Figure 6a–c) or brain cortex (Figure 6e–g). However, unexpectedly, we observed a decrease in staining for Iba1, a microglial activated marker, in serial sections of both tissues (Figure 6b middle panel, d,f middle panel, h). When we monitored microglial morphology, we observed microglia less ramified in 5xFAD/Rub−/− mice (Figure 7a), indicating more activated cells and a potential increase in neuroinflammation [53,54,55]. This observation was correlated with a significant increase in the expression of the pro-inflammatory cytokine TNF-α in brain cortex samples almost 10-times compared to those with a regular Rubicon expression (Figure 7c), with no differences in mRNA levels of other pro-inflammatory cytokines, such as IL-6 or TGF-β1 (Figure 7c). In hippocampus samples, we did not observe any difference in the expression of these cytokines (Figure 7b), suggesting a disconnection between amyloid β extracellular levels and neuroinflammation. Altogether, our results show that Rubicon loss did not change the number of neurons or astrocytes in the hippocampus or brain cortex of 5xFAD mice. Still, we did observe a change in microglia staining and morphology in brain cortex tissues from 5xFAD lacking Rubicon, which was correlated with an augmented expression of the TNF-α cytokine.

### 3.5. Autophagy Markers in 5xFAD Lacking Rubicon

**Figure 8:**
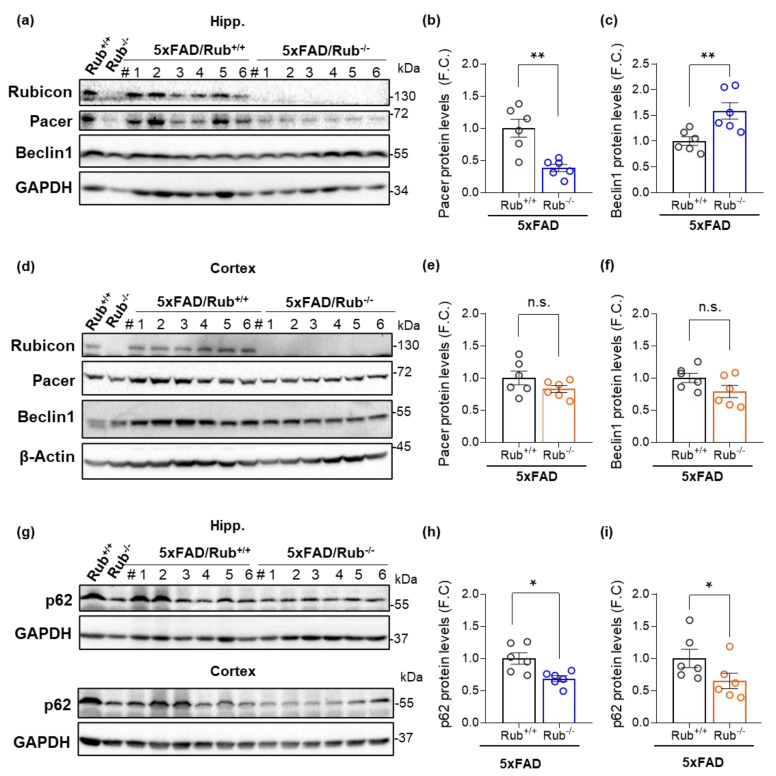
Autophagy markers in the hippocampus and cortex from 5xFAD lacking Rubicon. (**a**–**c**) Hippocampus; protein levels of Rubicon, Pacer, and Beclin1 were analyzed by Western blot in the hippocampus of mice with 5xFAD (5xFAD/Rub+/+) and 5xFAD lacking Rubicon (5xFAD/Rub−/−) at six months of age; (**b**,**c**) Bar graphs indicate the quantitative densitometry of the Pacer protein (**b**) and Beclin1 (**c**). (**d**–**f**) Brain cortex; protein levels of Rubicon, Pacer, and Beclin1 in the cortex of the same mice. #, number of independent animals. (**e**,**f**) Bar graphs indicate the quantitative densitometry of the Pacer protein (**e**) and Beclin1 (**f**). (**g**–**i**) Protein levels of p62 were analyzed by Western blot in the hippocampus ((**g**), upper panel) and in the cortex ((**g**), lower panel) of mice with 5xFAD (5xFAD/Rub+/+) and 5xFAD lacking Rubicon (5xFAD/Rub−/−) at six months of age; (**h**,**i**) Bar graphs indicate the quantitative densitometry of the p62 in the hippocampus (**h**) and cortex (**i**). β-Actin and GAPDH expression were monitored as loading controls. FC, fold change. Data are shown as means ± SEM (*n* = 6). * *p* < 0.05; ** *p* < 0.01 as determined by the Mann-Whitney *t*-test, n.s., no significance.

To address a potential correlation between amyloid β burden and autophagy activity in the mouse CNS, we assessed autophagy markers in the hippocampus, brain cortex, and lateral amygdala in 5xFAD mice with regular Rubicon expression (5xFAD/Rub+/+) and 5xFAD mice lacking Rubicon (5xFAD/Rub−/−) by Western blot. Interestingly, in hippocampus samples from 5xFAD/Rub−/− mice, we observed a 65% decrease in Pacer protein levels compared to 5xFAD/Rub+/+ mice (Figure 8a,b), a protein reported being inhibited by Rubicon [22,42,56]. We also observed the same phenomenon in the hippocampus of Rubicon KO mice compared to wild-type mice (Figure 8a, first two lanes). However, it was not observed in the brain cortex (Figure 8d) or basolateral amygdala samples (Supplementary Figure S5c,d). Beclin1 levels, on the other hand, were increased in the hippocampus from 5xFAD mice lacking Rubicon (Figure 8a,c), with no significant differences in brain cortex (Figure 8d,f) or amygdala samples (Supplementary Figure S5c,e). In hippocampus and cortex tissues, the levels of p62 were significantly decreased in 5xFAD/Rub−/− mice compared to 5xFAD/Rub+/+ (Figure 8g–i), suggesting an overall increase in autophagy activity in these regions. However, p62 levels were significantly increased in the basolateral amygdala from 5xFAD/Rub−/− (Supplementary Figure S5c,f). These results suggest Rubicon deficiency affects, in a differential manner, the CNS regions analyzed. Furthermore, while hippocampal Pacer is highly dependent on Rubicon levels, the absence of Rubicon seems not to affect Pacer in the brain cortex and amygdala. Decreased levels of Rubicon are correlated with a reduction in p62 accumulation in hippocampus and brain cortex samples.

### 3.6. APP-Expressing Neuro2A Cells Deficient in Rubicon Release more APP/Amyloid β

**Figure 9:**
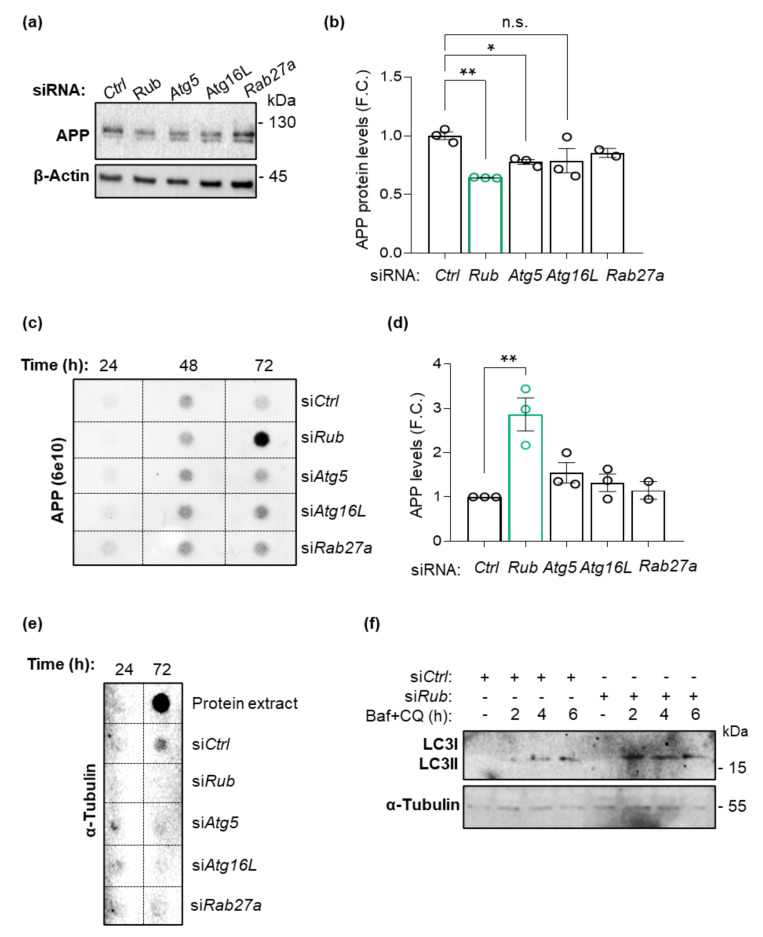
Neuro2A cells expressing human APP-EGFP lacking Rubicon release more APP species to the culture media. (**a**) Neuro2A cells expressing APP-GFP were transfected with siRNA against the autophagy proteins Rubicon (si*Rub*), Atg5 (si*Atg5*), and Atg16L (si*Atg16L*), an exosomal Rab, Rab27a (si*Rab27a*) or control scrambled (si*Ctrl*). Protein extracts were analyzed by Western blot using an anti-APP/amyloid β antibody (6E10). (**b**) Densitometric quantification of APP protein levels from protein extracts for each knockdown cell line. β-Actin expression was monitored as a loading control. (**c**) Culture media were collected 24, 48, and 72 h after transfection and analyzed by dot blot using the anti-APP/amyloid β antibody (6E10) and quantified (**d**). FC, fold change. (**e**) PVDF membranes containing culture media from each siRNA cell line plus a control with protein extract in RIPA 1x were incubated with α-Tubulin. (**f**) Autophagy flux in cells si*Rub* was performed using Bafilomycin A1 (25 nM) and Chloroquine (25 µM) (Baf + CQ) as lysosome inhibitors for 2, 4, and 6 h before preparing protein extracts. Data are shown as means ± SEM. * *p* < 0.05; ** *p* < 0.01 as determined by repeated-measures ANOVA, n.s., no significance.

The role of autophagy on APP and amyloid β levels has been highly discussed in the AD field. APP is present in membranes from endosomes and autophagosomes, being a target for lysosomal clearance via autophagy [9,14,57]. However, classical machinery autophagy components participate in other processes independently of autophagy. The role of Rubicon in APP or amyloid β species levels was explored in microglial-dependent LAP or LANDO processes. However, a contribution of neuronal Rubicon to APP homeostasis was not explored. To discriminate the impact of Rubicon-dependent autophagy on the levels of APP/amyloid β in a neuronal background, we generated neuron-like cells expressing human APP lacking Rubicon. Neuroblastoma (Neuro2A) cells expressing human APP-EGFP were transfected with a pool of siRNA targeting Rubicon (siRub) or scrambled siRNA control (siCtrl, control). After 72 h of transfection, protein extracts were prepared for Western blot. We found a decrease in APP levels in protein extracts in cells deficient for Rubicon compared to control siRNA (Figure 9a,b), suggesting that increased autophagy activity reduces intracellular APP (Figure 9f). To validate the effect of autophagy on APP levels in these cells, we promoted the deficiency of Atg5 (siAtg5) or Atg16L (siAtg16L), two proteins from the autophagy pathway essential for autophagosome generation [15,16,17]. Unexpectedly, we also observed a significant reduction in APP levels in cells lacking Atg5 (Figure 9a,b). While Rubicon knockdown stimulates autophagy activity (Figure 9f), siAtg5 cells present a reduction in its activity (Supplementary Figure S8b). To understand these results and correlate with increased amyloid β species burden observed in hippocampus from 5xFAD mice lacking Rubicon, we evaluated the conditioned media from the Neuro2A cells after 24, 48, or 72 h of siRNAs transfection. Intriguingly, we found an accumulation of extracellular APP in the medium derived from cells deficient for Rubicon after 72 h (Figure 9c,d). In contrast, siAtg5 or siAtg16L cells did not change the APP species release. Hence, our results propose that Rubicon inhibits APP/amyloid β release or secretion in an Atg5-independent process. To ask if this release is mediated by exosomes secretion, we knocked down Rab27a (siRab27a), a small protein required for exosome secretion [58]. As shown in Figure 9, the knockdown of Rab27a did not influence APP levels in protein extracts or the conditioned media (Figure 9a–d), suggesting that APP/amyloid β was released independently of exosome secretion. To control that cell content or debris was not released to the conditioned media in siRNA lines, we performed a dot blot assay using whole-cell extracts. We observed a clear signal for α-Tubulin only in the control protein extracts, confirming the absence of cellular debris by toxicity on conditioned media (Figure 9e).

Although siRub cells show increased LC3-II levels in an autophagy flux analysis using lysosomal inhibitors (Figure 9f), our data suggest that the increased levels of APP in the conditioned media are not dependent on canonical Atg5 dependent of autophagy. Furthermore, our results propose an uncharacterized role of Rubicon in neuronal APP/amyloid β secretion, independent of its previously published role in microglial phagocytosis. The increased amyloid β burden observed in the hippocampus from 5xFAD mice lacking Rubicon could result from a synergistic effect of reduced microglial LAP/LANDO activity combined with increased neuronal APP/amyloid β release.

## 4. Discussion

Autophagy occurs at basal levels in virtually all eukaryotic cells. In neurons, autophagy presents an essential role in maintaining cellular homeostasis and responding to stress stimulus conditions. In parallel, several autophagy machinery components could participate in intracellular processes in an autophagy-independent way. Rubicon is one of the few inhibitory proteins of the autophagy pathway and a positive member of alternative phagocytosis processes in phagocytic cells, such as microglia. In a complex tissue, such as CNS, shaped by different types of cells, discriminating which processes are more prompt to occur in a determined context is essential for understanding disease pathogenesis and designing future treatments. In the present study, we found a marked neuronal pattern of expression of Rubicon in CNS from mice and postmortem samples from AD patients, as previously reported by others and ourselves in mice [33,42,52]. However, by single-cell transcriptomic analysis, Rubicon was also detected in other CNS cells, including oligodendrocytes, astrocytes, and microglia [8].

Rubicon levels were significantly increased in hiPSC clones from an AD patient at the early-to-moderate stage of the disease, confirmed in postmortem frontal cortex samples from AD patients from advanced disease progression. Elevated levels of Rubicon have been previously correlated with a reduction in autophagy activity in several cells, including neurons [28], cardiomyocytes [59,60], hepatocytes [61], adipocytes [62,63], and retinal epithelium [64]. Crescent Rubicon levels during aging have been associated with the decline in autophagy activity, arising as one of the potential target molecules in aging [28,29,65]. A recent study reported some components of this potential pathway [29]. Researchers identified the transcription factor MondoA, which promotes longevity in *C. elegans* through caloric restriction or germinal removal [66,67], as a brake for senescence by partially reducing Rubicon levels [29]. In mammalian cells treated with siRNA against MondoA, researchers found Rubicon as one of the overexpressed DEGs (differentiated expressed genes) by RNA-sequencing analysis, together with a decrease in Atg7 and Atg12 expression, resulting in autophagy inhibition [29]. The decline in MondoA during aging would allow the increase in Rubicon levels, potentiating the autophagy inhibition. Of note, MondoA is a glucose sensor, which could situate Rubicon as a potential modulator of the metabolism and glucose control [68].

Mechanistically, Rubicon inhibits Pacer and UVRAG in the PI3KC3, preventing endosome and autophagosome maturation [22,23,24]. A mutation in the C-terminal domain of Rubicon was identified in a patient with a recessive form of ataxia, a neurological disorder caused by the lack of voluntary coordination of muscle movements. This mutation promotes the Rubicon to switch from endosome/lysosome to a diffuse cellular pattern [50,51], failing to follow its function, showing the importance of Rubicon to CNS homeostasis. Indeed, the mouse model lacking Rubicon used in the present work was generated by deleting the region associated with this mutation in mice. However, no movement difficulties were observed in Rubicon KO mice in [30] and in our work. 

Interestingly, our AD mouse model lacking Rubicon showed different outcomes depending on the CNS region analyzed. We performed anteroposterior serial slices from experimental brain mice to analyze the entire hippocampus and layer V from the brain cortex. In the hippocampus, mice lacking Rubicon present a significative rise in the amyloid β burden, not correlated with a loss of neurons or impairment in the MWM memory test. However, we observed a significant depletion in Pacer levels in hippocampus samples from mice lacking Rubicon and 5xFAD/Rub−/− mice, suggesting a dependency or compensatory regulation between both proteins in this region. No differences were observed between groups in Pacer levels in the brain cortex and basolateral amygdala. Alternatively, we observe an increase in Beclin1 levels in hippocampal tissues. Beclin1 contributes to the endocytosis and autophagy in neurons and is essential to the function and viability of hippocampal neurons [69,70]. The rise of Beclin1 in 5xFAD/Rub−/− mice hippocampus could partially contribute to the lack of differences in the spatial memory MWM test or neuronal loss between groups, despite an augmented plaque’s burden in the hippocampus. Indeed, a reduction in Beclin1 levels, specifically in hippocampal neurons, abolishes memory formation in mice [70]. The same work showed that old mice (16 months old) treated with systemic blood injections from young mice recovered their memory loss. This effect was absent in mice lacking Beclin1 in hippocampal neurons [70]. On the other hand, p62 levels were reduced in hippocampi and brain cortex samples from 5xFAD/Rub−/− mice, indicating a potential overall increase in autophagy flux in these regions. However, in the basal lateral amygdala, a region reported to be atrophied in severe AD patients and harboring extensive Aβ plaques and NFT [71,72,73], there were no differences in the levels of Pacer or Beclin1 and a surprising increase in p62 in mice lacking Rubicon. Few studies have explored the differences in autophagy activity in CNS regions that could explain the different outcomes between brain regions. A work on this topic proposes that the murine hippocampus is more likely to be affected in neurodegenerative diseases due to reduced basal autophagy machinery able to cope with increased protein aggregates [74]. More detailed studies in specific regions and specific autophagy-related processes would help to clarify these differences. In summary, our results suggest that neurons from different regions may have different Rubicon requirements concerning the regulation of autophagy and other roles of the protein. 

Besides negatively regulating autophagy, Rubicon participates as a positive component in specialized phagocytosis (LAP) and endocytosis (LANDO) processes in some phagocytic cells, including microglia [30,31,75]. In LAP, Rubicon was shown to recruit PI3KC3 to single-membrane phagosomes, following the recruitment of the NOX2 complex, thereby promoting ROS production and, finally, recruiting LC3 [30]. The role of Rubicon in these processes has been associated with inflammation and immune response. Indeed, a work proposed that extracellular amyloid β levels are dependent on microglial LAP, as demonstrated by increased amyloid β burden in the hippocampus of 5xFAD mice lacking Rubicon [31], as in our data. Although the authors did not evaluate Rubicon localization or cell-type expression, their results were checked using a cell-type-specific Rubicon KO mouse model for microglia. We evaluated microglial activation in both groups of 5xFAD mice. Although the total fluorescence is decreased in 5xFAD lacking Rubicon, the microglial morphology in those mice is characteristic of active microglia, showing a decrease in cellular ramification and an ameboid shape [53,54,55]. This observation was correlated with a significant increase in the expression of the pro-inflammatory cytokine TNF-α, close to ten-times in the brain cortex compared to 5xFAD wild-type to Rubicon, but not in the hippocampus. These data suggest that, at least in a local microenvironment, increased neuroinflammation modulators do not directly affect amyloid β release or vice-versa. 

APP is a transmembrane protein endocytosed and processed in the autophagy and endolysosome systems. We hypothesized that increased levels of Rubicon could prevent APP degradation in lysosomes, a common final fate for cargos of endocytosis or autophagy pathways. On the other hand, decreasing Rubicon levels were expected to activate autophagy and promote lysosomal degradation of its cargos. To further characterize the role of the Rubicon on APP levels in a neuronal context, we promoted Rubicon deficiency in NeuroA2 cells expressing human APP. Analyses of APP/amyloid β species in whole protein extracts versus in the extracellular medium showed that the knockdown of Rubicon significantly increased extracellular APP/amyloid β, with a reduction in APP in protein extracts. In contrast, the knockdown of autophagy proteins Atg5, ATg16L, or the small GTPase mediating exosomal secretion, Rab27a, had no effect. These results propose a specific role of Rubicon in regulating APP/amyloid β extracellular release in an Atg5-independent process. Interestingly, these results reinforce a relationship between perturbations in core processes of proteostasis and protein secretion in AD pathogenesis [9,76]. The increased amyloid β burden observed in the hippocampus from 5xFAD mice lacking Rubicon could result from a synergistic effect of reduced microglial LAP activity combined with increased neuronal APP/amyloid β release.

## 5. Conclusions

In conclusion, our results suggest that the presence of Rubicon negatively regulates APP/amyloid β release to the extracellular medium. It remains to be investigated whether APP/amyloid β is a specific target of this type of regulatory role of Rubicon or if there are others. Similarly, future research will be needed to elucidate whether this function of Rubicon is associated with its reported roles in autophagy, endocytosis, phagocytosis [23,24,28,29,30], or an independent function from those pathways. Our work also proposes that this unprecedented action of Rubicon on APP/amyloid β release could define a new potential way to target AD and other similar diseases therapeutically. Our results amplify the understanding of the multifunctional protein Rubicon in the context of AD, including a new role for Rubicon as a negative regulator of APP/amyloid β release.

## Figures and Tables

**Table 1:** Patients’ information.

Donor	Gender	Disease Duration (Years)	CDR	Braak Stage
C1	Male	-	0	I
C2	Female	-	0	II
C3	Female	-	0	II
C4	Female	-	0	II
AD1	Female	6	3	IV
AD2	Female	6	3	IV
AD3	Male	6	3	IV
AD4	Male	10	3	IV

(C, controls; AD, Alzheimer’s disease patients).

## Data Availability

Not applicable.

## Supplementary Materials

The following supporting information can be downloaded at: https://www.mdpi.com/article/10.3390/cells11121860/s1, Supplementary Methods; Figure S1. Characterization of the hiPSC pluripotency and transcriptional profile showed increased levels of Rubicon in the cerebral cortex of AD transgenic mice by Western blot; Figure S2. 5xFAD and Rubicon-lacking mice genotyping; Figure S3. Control for antibodies employed for the human frontal brain samples immunofluorescence; Figure S4. Rubicon is located in neurons in hippocampal and brain cortex sections of wild-type mice (6 months old); Figure S5. Amyloid β burden presents no changes in basolateral amygdala sections from 5xFAD-lacking Rubicon mice at the symptomatic stage; Figure S6. No changes in neuron number depending on Rubicon levels; Figure S7. Rubicon levels did not influence the cognitive Morris water maze test in 5xFAD mice; Figure S8. Neuro2A cells expressing APP-GFP knockdown to Atg5, rab27A, and ATG16L.
