# Supplementary material for: Neuronal Rubicon Represses Extracellular APP/Amyloid β Deposition in Alzheimer’s Disease
Source: Cells. 2022 Jun 7;11(12):1860. doi: 10.3390/cells11121860 (PMC9221152; doi:10.3390/cells11121860)
Supplement: Supplementary file 1 [file cells-11-01860-s001.zip › cells-1730150-supplementary.pdf]

Supplementary Material and Methods

Cells immunofluorescence

For IF, hiPSCs were fixed in 4% PFA, permeabilized, blocked, and incubated with the following primary antibodies: anti-SOX2 (goat, 1:200, Santa Cruz Biotechnology); anti-OCT4 (mouse, 1:50, Santa Cruz Technology); and anti-Rubicon (rabbit, 1:1000, Thermo Fisher Scientific, PA5-38017). Secondary antibodies were anti-mouse or anti-rabbit Alexa 488 (1:500, Thermo Fisher Scientific, A11001 and A11008, respectively). ProLong Gold antifade with DAPI was used as a mounting medium (Life Technologies).

Mice genotyping

Genotyping was carried out by extracting DNA by the pierced ear tag method. The ear tissue was incubated with 600 μL of lysis buffer (50 mM Tris pH 8.0, 100 mM EDTA, 100 mM NaCl, and 1% SDS) and 6 μL of 10 mg/mL proteinase K for 16 h at 55°C followed by centrifugation for 5 min at 13000G. The supernatant was mixed with 500 μL of 100% isopropanol, and the DNA was observed as a white residue. The pellet was washed with 750 μL of 70% ethanol, resuspended in 50 μL of TE buffer (10 mM Tris-Cl pH 7.5, 1 mM EDTA) incubated for three hours at 55°C to facilitate homogenization. A conventional multiplex PCR was used to identify the transgenic animals from the 5xFAD colony. Primers used for the 5xFAD genotype were: APP 5′-AGG-ACT-GAC-CAC-TCG-ACC-AG-3' and 5′-CGG-GGG-TCT-AGT-TCT-GCA-T-3', and PSEN 5′-ATT-AGA-GAA-CGG-CAG-GAG-CA-3' and 5′-GCC-ATG-AGG-GCA-CTA-ATC-AT-3'. The PCR program used was one cycle of denaturation for 3 min at 95°C, 30 cycles of 30 sec at 95°C, 1 min at 57°C, 1 min at 72°C, and a final cycle of elongation for 10 min at 72°C. We used the following primers for the Rubicon KO colony: Rubicon 5′-AGC-AGAGGG-TTT-TAT-GCG-CT -3' and 5′-CCA-CCA-CAC-CCA-ACT-CTT-CA-3'. The PCR program to identify these animals was one cycle of denaturation for 3 min at 94°C, 35 cycles of 30 sec at 94°C, 30 sec at 56°C, 60 sec at 72°C, and a final cycle of elongation for 10 min at 72°C. The PCR product was digested with the EcoRI enzyme to recognize the generated alleles (Supplementary Figure 4a).

Morris water maze

The Morris Water Maze behavioral test was performed to assess the hippocampus-dependent memory function with the Anymaze software (Stoelting Co.). At six months, mice were placed in the pool and were taught to swim around the pool and find the hidden platform following spatial cues in the room for orientation for 1 minute. Age- and sex-matched animals (WT/$Rub^{+/+}$ or WT/$Rub^{-/-}$, n= 14 each; 5xFAD/$Rub^{+/+}$, n=23; and 5xFAD/$Rub^{-/-}$, n=20) were trained four times per day for four days. The time the subject needed to reach the platform was measured. On the fifth day, the platform was extracted, and the time the animal spent on the platform quadrant was measured. The distance traveled by the animals was also monitored.

References

Supplementary Figures Legends

Supplementary Figure S1. Characterization of the hiPSC and transcriptional profile of Rubicon. a. Representative confocal images of immunocytochemistry for SOX2 (red) and OCT4 (green) in **(b)** merged with Dapi (blue). **c.** The transcriptional profile of pluripotent markers *OCT4* and *SOX2* were determined by quantitative PCR, showing similar levels in control and AD-hiPSCs (n=3 to 7). 18S expression was monitored as a housekeeping gene. **d.** RUBICON mRNA levels in control and AD-hiPSCs (n=9). Data shown are mean ± SEM. The statistical significance was defined as $P<0.05$ by the Mann-Whitney test, n.s., no significative.

Supplementary Figure S2. 5xFAD and Rubicon-lacking mice genotyping. a. PCR products in agarose gel showing genomic fragments to check human APP and PS1 from 5xFAD mice (lower image) and Rubicon fragments after enzyme digestion. **b.** Brain cortex protein extracts from one wild-type ($Rub^{+/+}$) and two mice lacking Rubicon ($Rub^{-/-}$) were detected by Western blot using an anti-Rubicon antibody. **c.** Confocal image from brain cortex from wild-type and KO Rubicon mice detected by an anti-Rubicon antibody (red) by immunofluorescence. Nuclei are stained with DAPI. Scale bar: 20 μm.

Supplementary Figure S3. Control for antibodies employed for the human frontal brain samples immunofluorescence. To evaluate unspecific antibody staining, postmortem samples were stained for nuclei with Dapi and secondary antibodies for RUBICON (rabbit) and NeuN (mouse) without incubation with primary antibodies.

Supplementary Figure S4. Rubicon is located in neurons in hippocampal and brain cortex sections of wild-type mice (6 months old). Z-stack of confocal images detecting Rubicon (red), the neuron marker NeuN or the astrocytic marker GFAP (green) by immunofluorescence in the hippocampus (**a** and **b**) and brain cortex (**c** and **d**). Nuclei are stained with DAPI. Images are representative of at least four animals. Scale bar: 50 μm; inset: 20 μm.

Supplementary Figure S5. Amyloid β burden presents no changes in basolateral amygdala sections from 5xFAD lacking Rubicon mice at the symptomatic stage. a. Representative Z-stack of immunocytochemical confocal images of amyloid β plaque detection (4G8, red) in the basolateral amygdala of 5xFAD mice with regular expression of Rubicon (5xFAD/$Rub^{+/+}$) on the left and mice lacking Rubicon (5xFAD/$Rub^{-/-}$). Images are representative of at least four animals for each genotype. **b.** Quantification of the intensity of fluorescence of 4G8 burden in sections of the basolateral amygdala. Data are mean ± SEM. $P<0.05$ as determined by the Mann-Whitney test. **c.** Protein levels of Rubicon, Pacer, Beclin1, and p62 were analyzed by Western blot in mice's basolateral amygdala in 5xFAD/$Rub^{+/+}$ and 5xFAD/$Rub^{-/-}$ at six months of age. **d-f**. Bar graphs indicate the quantitative densitometry of the Pacer protein **(d)**, Beclin1 **(e)**, and p62 **(f)**. GAPDH expression was monitored as a loading control. Data are shown as means ± SEM (n=4 and 5). $^*P < 0.05$ as determined by the Mann-Whitney t-test, n.s., no significative.

Supplementary Figure S6. No changes in neurons number depending on Rubicon levels. Representative immunocytochemistry images for detecting neurons (violet cresyl) in the hippocampus of 5xFAD mice with and without expression of Rubicon (5xFAD/$Rub^{+/+}$; 5xFAD/$Rub^{-/-}$). Inset: showing a zoom from the white square.

Supplementary Figure S7. Rubicon levels did not influence the cognitive Morris water maze test in 5xFAD mice. a. Morris water maze test (MWM) to test hippocampus-dependent memory in mice was performed in wild-type (WT/$Rub^{+/+}$, n=14), wild-type lacking Rubicon (WT/$Rub^{-/-}$, n=14), and

5xFAD mice from both genotyping (5xFAD/Rub$^{+/+}$, n=23, and 5xFAD/Rub$^{-/-}$, n=20). After four days, 5xFAD/Rub$^{+/+}$ presented increased latency for the first entry to the platform compared to WT mice. However, no differences were observed between mice lacking Rubicon. **b.** Distance traveled by four groups measured in meters (m). Data are mean ± SEM. **P<0.01 as determined by repeated-measures ANOVA n.s., no significative.

Supplementary Figure S8. Neuro2A cells expressing APP-GFP knockdown to Atg5, rab27A, and ATG16L. a. si*Rub* knockdown verification by real-time quantitative PCR (n=4). **b.** Neuro2A cells were transfected with si*RNA* against the autophagy protein Atg5 (si*Atg5*), and the autophagy flux was performed using Bafilomycin A1 (25nM) and Chloroquine (25 μM) (Baf+CQ) as lysosome inhibitors for 2, 4, and 6 hours before preparing protein extracts. Anti-atg5 was used to verify the knockdown. **c.** si*rub27a* knockdown verification by real-time quantitative PCR (n=3). **d.** Neuro2A cells were transfected with si*RNA* against the autophagy protein Atg16L (si*Atg16L*). The autophagy flux was performed using the same lysosome inhibitors described in **a** for 6 hours before preparing protein extracts. Data are shown as means ± SEM. *P < 0.05 and **P<0.01 as determined by the Mann-Whitney t-test, n.s., no significative.

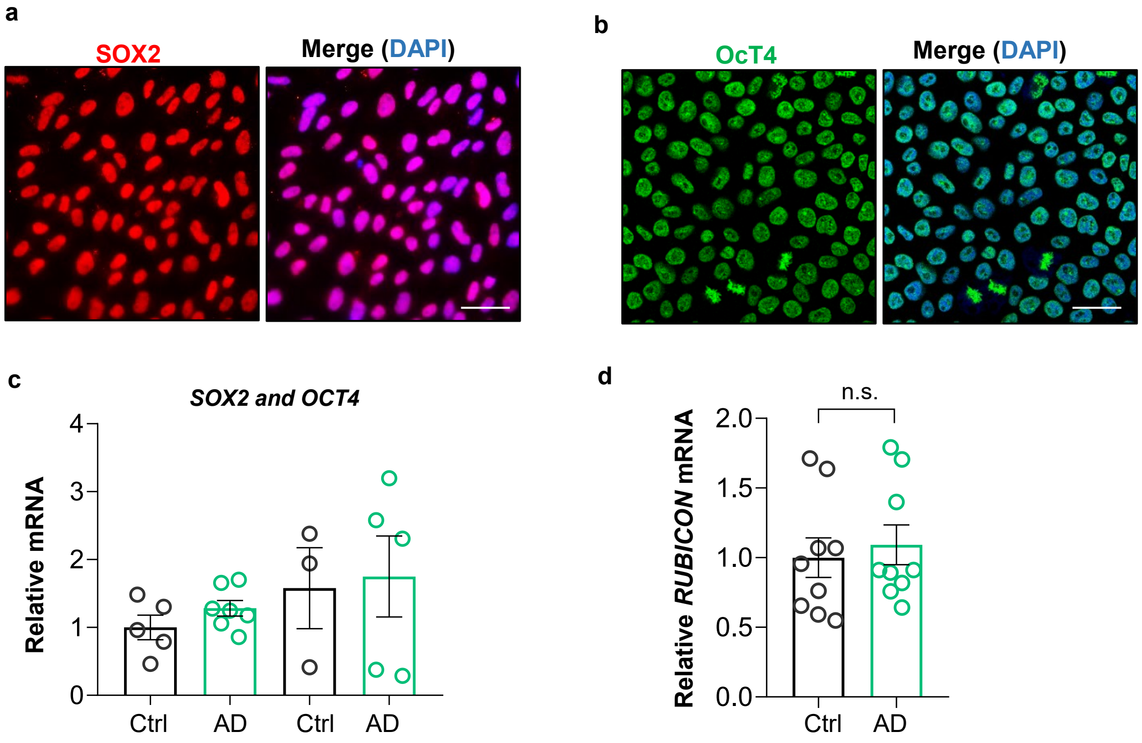

Supplementary Figure S1

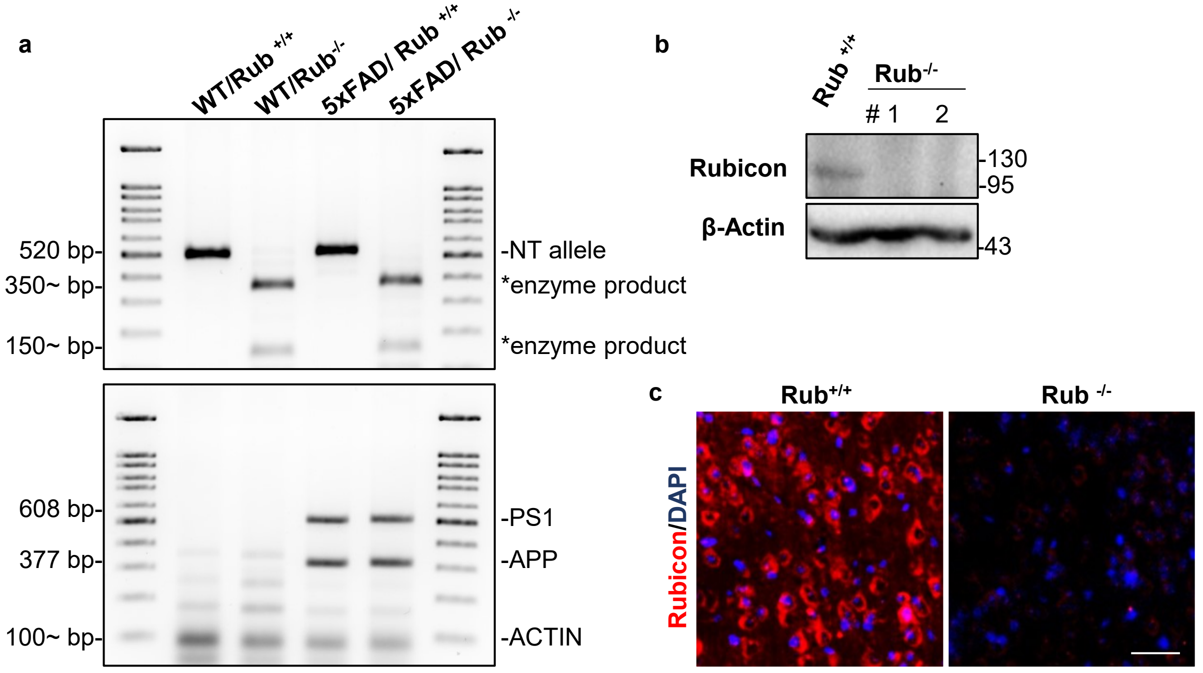

Supplementary Figure S2

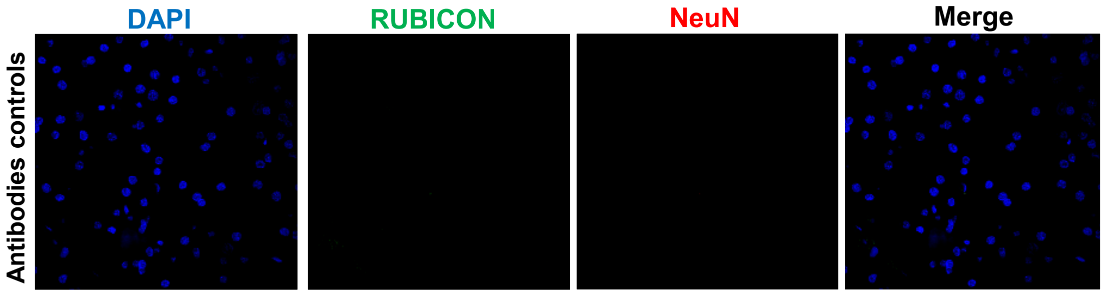

Supplementary Figure S3

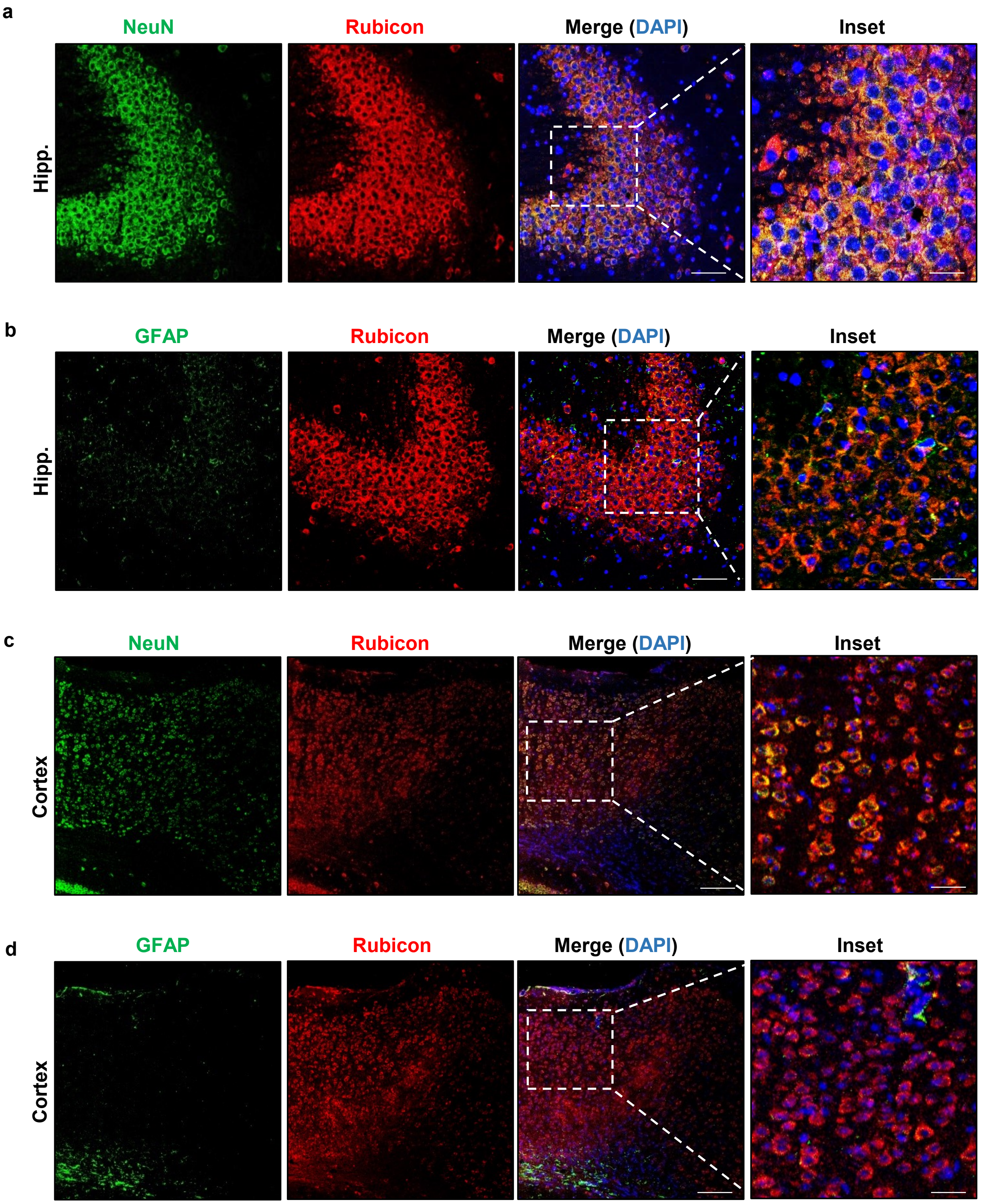

Supplementary Figure S4

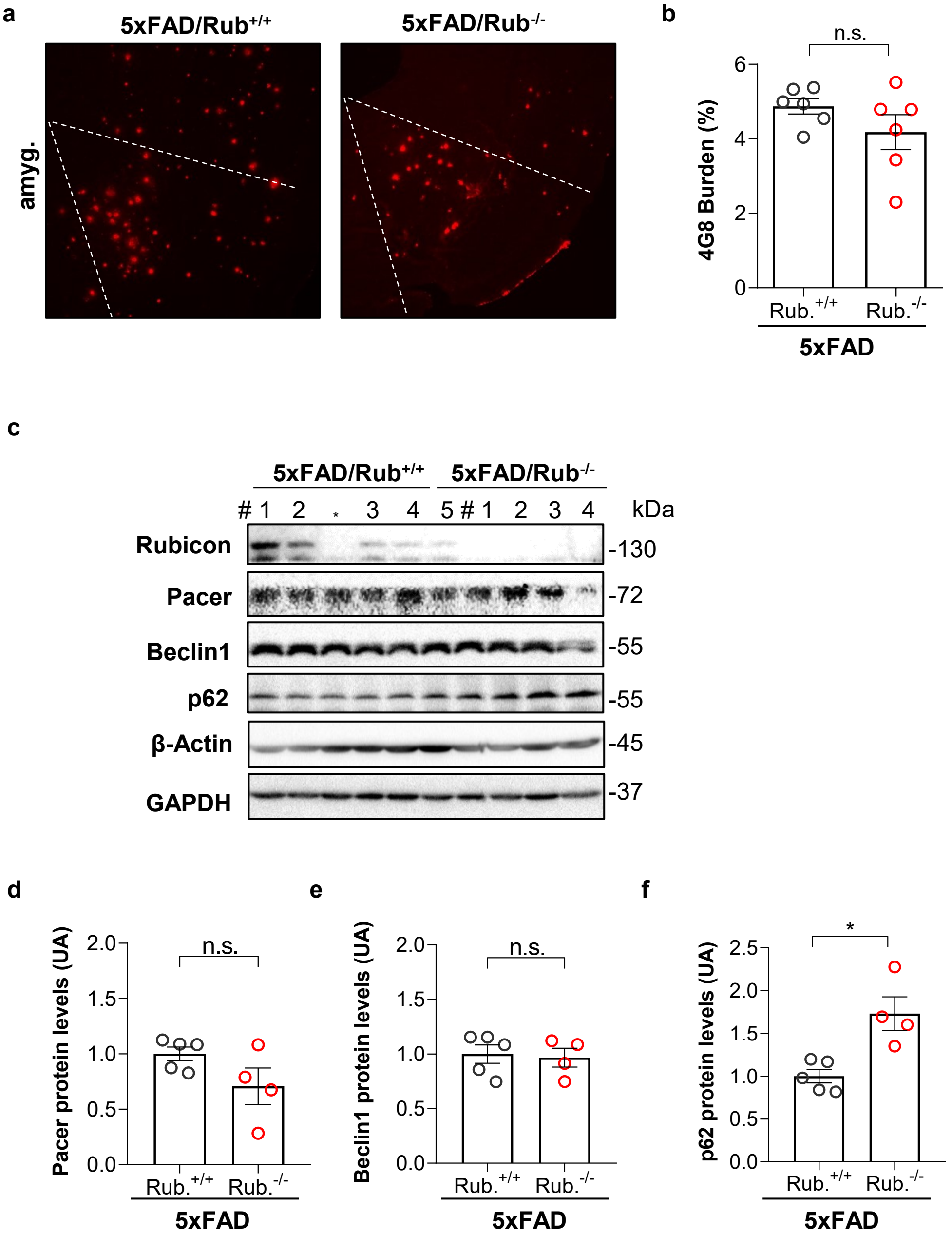

Supplementary Figure S5

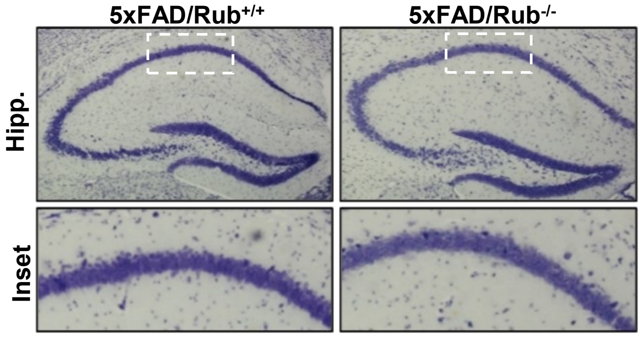

Supplementary Figure S6

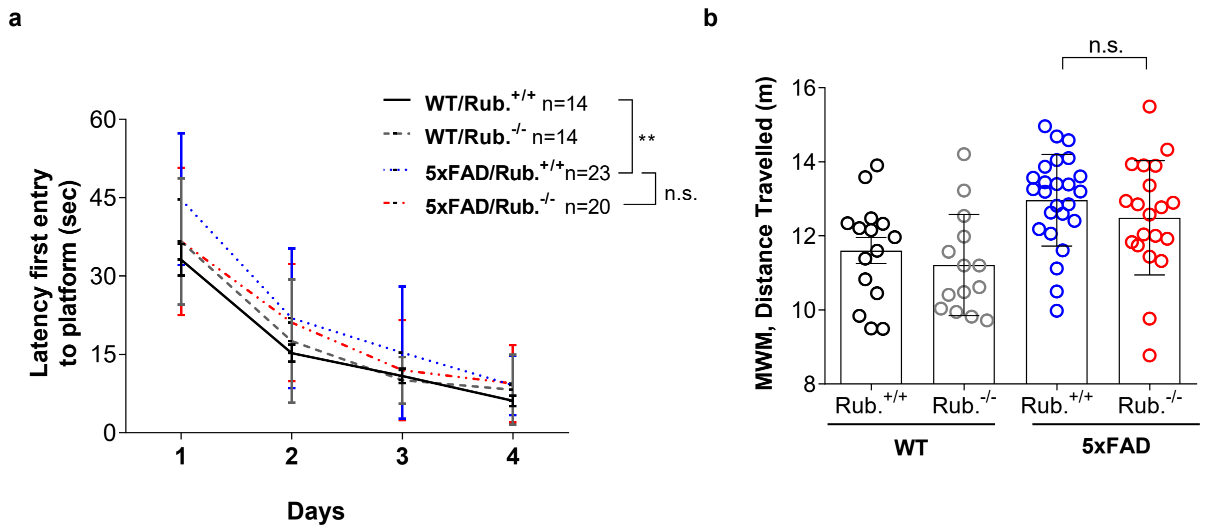

Supplementary Figure S7

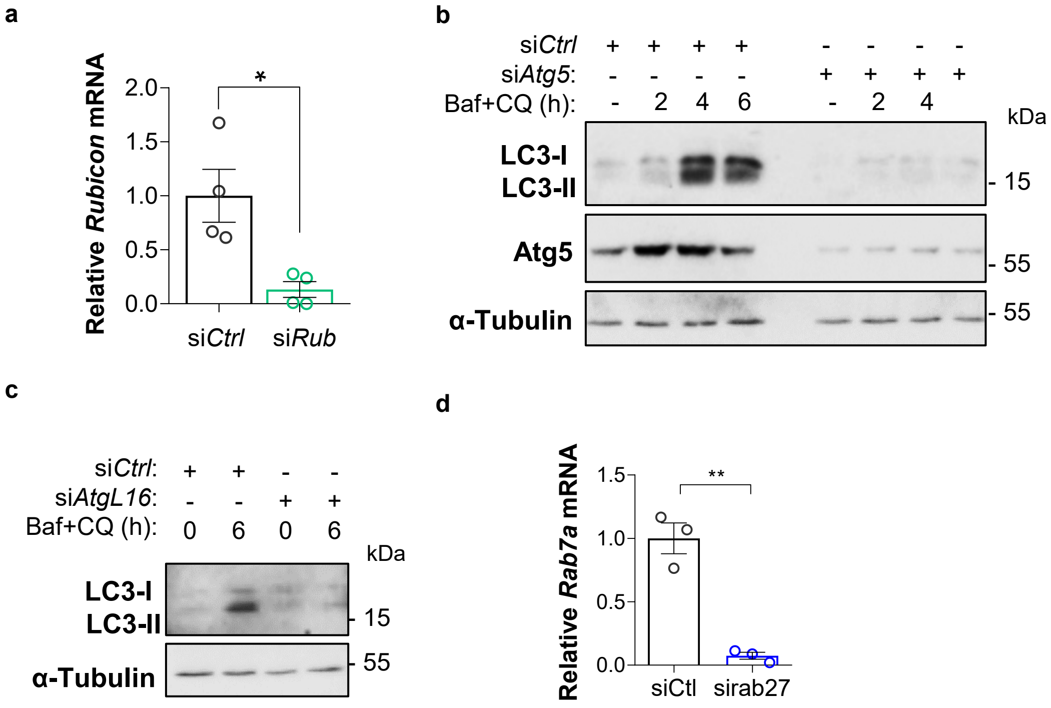

Supplementary Figure S8
